# Using Bus Ticketing Big Data to Investigate the Behaviors of the Population Flow of Chinese Suburban Residents in the Post-COVID-19 Phase

**DOI:** 10.3390/ijerph18116066

**Published:** 2021-06-04

**Authors:** Yanbing Bai, Lu Sun, Haoyu Liu, Chao Xie

**Affiliations:** 1 Center for Applied Statistics, School of Statistics, Renmin University of China, Beijing 100872, China; ybbai@ruc.edu.cn (Y.B.); 2017201665@ruc.edu.cn (H.L.); 2 School of Statistics, Southwestern University of Finance and Economics, Chengdu 611130, China; 3 China Transport Information Co., Ltd., Beijing 100007, China; xiechao@transinfo.com.cn; 4 China Transport Telecommunications and Information Center, Beijing 100011, China

**Keywords:** bus ticketing big data, spark distributed computing, population flow, chinese suburban residents, post-COVID-19 phases

## Abstract

Large-scale population movements can turn local diseases into widespread epidemics. Grasping the characteristic of the population flow in the context of the COVID-19 is of great significance for providing information to epidemiology and formulating scientific and reasonable prevention and control policies. Especially in the post-COVID-19 phase, it is essential to maintain the achievement of the fight against the epidemic. Previous research focuses on flight and railway passenger travel behavior and patterns, but China also has numerous suburban residents with a not-high economic level; investigating their travel behaviors is significant for national stability. However, estimating the impacts of the COVID-19 for suburban residents’ travel behaviors remains challenging because of lacking apposite data. Here we submit bus ticketing data including approximately 26,000,000 records from April 2020–August 2020 for 2705 stations. Our results indicate that Suburban residents in Chinese Southern regions are more likely to travel by bus, and travel frequency is higher. Associated with the economic level, we find that residents in the economically developed region more likely to travel or carry out various social activities. Considering from the perspective of the traveling crowd, we find that men and young people are easier to travel by bus; however, they are exactly the main workforce. The indication of our findings is that suburban residents’ travel behavior is affected profoundly by economy and consistent with the inherent behavior patterns before the COVID-19 outbreak. We use typical regions as verification and it is indeed the case.

## 1. Introduction

The immediate impact of the COVID-19 outbreak has brought significant problems to people’s health, safety, and critical supply chains, which has produced many other social and economic impacts [1]. With the suspension of the Chinese industry, numerous global supply chains were disrupted, and the prices of raw materials, stocks, and oil fallen sharply [2]. To control the spread of COVID-19, the government confined the travel behaviors, and a large number of flights were canceled [2], the public transportation fallen into a plight. Grasping the travel behaviors disturbed by public emergencies of the COVID-19 epidemic is crucial to formulating scientific and reasonable prevention and control policies. Moreover, large-scale population movements can turn local diseases into widespread epidemics [3,4], so researching the population flow is also of great significance for providing information to epidemiology.

The COVID-19 epidemic can be divided into three phases, with previous literature determining the three phases are: the early phase of COVID-19, the COVID-19 pandemic, and the post-COVID-19 phase. Regarding the early phase of the COVID-19 epidemic, people mainly studied and paid attention to the epidemiological and clinical characteristics of COVID-19 [5,6,7], as well as risk perception and preventive measures for COVID-19 [8,9,10]. Muto et al. [11] have taken Japan as an example to understand the changes in people’s social behavior and their responses to government calls. Respecting the pandemic of the COVID-19, scholars turned concerns on the passive impact of the COVID-19 on the education [12], economy [2] and many other fields, as well as policy amendments [13] and the turning point prediction [14,15,16]. Of the three phases, the post-COVID-19 phase has the least research and investigation.

The post-COVID-19 phase refers to 29 April, 2020, to the present, judging by the White Paper on “Fighting COVID-19 China in Action”, when the prevention and control of the COVID-19 in the whole country have become routine. The economy, industry, and transportation are all gradually recovering and approaching the patterns before the epidemic. In particular, we can consider the post-COVID-19 phase as the “period of recovery”. Understanding the various social behaviors of people at this phase is crucial to the reconstruction and recovery process after the COVID-19 epidemic, formulating scientific and reasonable policies to maintain the current anti-epidemic results and helping people adjust to their everyday lives quickly and satisfactorily.

Issues worthy of taking attention and investigating in the post-COVID-19 phase have been discoursed strongly in the literature, including health strategies, economic recovery and lifestyle changes, etc. The relevant literature has pointed out that [17] the post-COVID-19 phase is the time to understand the future development of the COVID-19 and the consequences for survivors. Understanding the potential acute care needs of recovered COVID-19 patients is significant, promoting a reasonable health strategy. Regarding economic recovery, scholars [18] took the European as an example and found that although emergency financial support can solve the short-term demand and supply problem of the capital chain, it may harm the relational market. Therefore, the scholars proposed that economic recovery should be carried out in three steps: The first stage is emergency liquidity; the second stage is solvency support; the third stage is to reboot the economy. The literature considering [19] on the changes in the lifestyle and travel behavior of Chinese tourists in the post-COVID-19 phase found that new forms of travel that reduce exposure, such as free travel, independent travel, and health tourism, are becoming more and more popular. Health and wellness tourism generally refers to being close to nature, visiting mountains, rivers, and lakes. The purpose is to relax and avoid crowds gathering. Now bathing in hot springs has become a trendy way to relax. Tourism and transportation, as the most sensitive industries to the COVID-19, deserve more attention [20,21]. Unlike the energy industry, tourism is less necessary and highly sensitive to emergencies [20], so changes in travel behaviors and patterns can be used as a “barometer” of how the COVID-19 epidemic is evolving.

Regarding the impact of emergencies on travel behaviors and patterns, some scholars studied the recovery situation after the SARS virus outbreak [22]. It is found that although the travel industry lacks resistance to short-term crises, it has strong resilience. The indicators of the travel industry declined severely in March 2003 when the SARS outbreak, but the epidemic was under control rapidly, and the number of new infections dropped to zero in June, then the transportation and travel industry approximately recovered in July. It only took about one month rebooting from the impact epidemic.

Some scholars had made efforts to investigate the travel behavior and recovery in the post-COVID-19. Ivanova et al. [23] found that most of the 974 respondents were willing to travel after liberalizing restrictions, and their preferred travel destination was rural areas. The survey also indicated that female and older respondents have higher requirements for safety and health than male and younger respondents, and their travel attitude is relatively conservative.

Nguyen et al. [24] also found that different groups respond differently to the COVID-19. Crisis-sensitive travelers are more likely to be affected by the external environment and travel with a more conservative attitude. However, crisis-resistant travelers are almost the same as the early phase before the COVID-19 outbreak. The main characteristics distinguishing crisis-sensitive travelers and crisis-resistant travelers include gender, age, education level, and family income. The elderly and higher-income people are more sensitive to the crisis. On the contrary, the younger and low-income people are more resistant to the crisis. Actually, it is just the appearance. Younger and low-income people are not easily affected by external conditions, more likely because they are captive and have a limited capacity to do differently, so they have to maintain the present situation. However, this is still a social phenomenon and population characteristic that is very worthy of attention. Jain et al. [25] also found that travel behaviors would be significantly affected by income.

The previous results are obtained through questionnaire surveys, and the sample size (number of respondents) is less than 1000. The results are easily affected by the respondents’ subjective thoughts and the sampling irrationality, making the results biased. To avoid unreliable results due to few samples or unstable sampling, we use extensive data analysis to obtain more scientific, reasonable, and stable results, which are more convincing.

Most suburban residents are middle-income or low-income groups [26,27], and their travel behaviors are more casual and uncontrolled. China, as a country with numerous suburban residents, investigating their travel behaviors is essential for protecting the achievement of fighting the COVID-19 epidemic and maintaining national stability.

However, estimating the impacts of the COVID-19 for suburban respondents’ travel behaviors remains challenging because of lacking apposite data. Suburban residents may seldom take airplanes, high-speed rails, and other high-fare vehicles due to limited income or other reasons. It results in the flight and railway data not fully cover the population we want to investigate. So we presented the ticketing data of long-distance buses to make up for this shortcoming.

The long-distance bus ticketing data has been demonstrated to be valid at reflecting the travel behavior of suburban residents, which has been expounded by statistical reports of China’s authoritative transportation agency [28]. The reports shows that the highest proportion of travel reasons is commuting, and according to the residential structure of Chinese residents and the arrangement of bus routes, most bus passengers indeed live in the suburbs, and the route arrangement tends to be in the suburbs or urban fringe to connect the inaccessible places by planes and trains. Apart from this, the research of Gray et al. [29] also could verify this phenomenon. They found that suburban residents would choose to travel by bus/coach or train to reduce costs. Although they want people to pay more attention to the role of the train in suburban travel, they deem that people significantly ignored the role of train travel. However, it also just shows that the status of bus travel in suburban areas can not be ignored.

Investigations and studies on long-distance bus travel behavior and patterns are few. Most of them focus on urban traffic, or a few years ago [30]. There is less literature about bus travel behavior around the whole country after the outbreak of COVID-19. So it makes our research necessary and urgent.

In this paper, we investigated suburban residents’ travel behaviors and patterns across the country from the spatial dimension. We found that the travel behaviors of suburban residents in the post-COVID-19 phase are mainly affected by economic and historical-cultural elements rather than COVID-19. Suburban respondents’ travel behavior is almost casual and not as conservative as we imagined. In particular, we selected specific regions as verification (3.3) to prove that this is indeed the case.

Our innovation points are as follows:

(1) Using the bus ticketing big data are more reliable and could illustrate the travel patterns of suburban residents well. Make up for the lack of apposite data for suburban residents.
(2) Understanding the spatial characteristics of travel behaviors and patterns around China, not limited to just one city.
(3) Grasping suburban residents’ travel behavior and travel patterns is crucial to maintaining the current anti-COVID-19 results and national stability.

## 2. Materials and Methods

### 2.1. Data Introduction

The main data we use is bus ticketing data. Baidu API is only used as an aid to match location information. The bus data comes from China’s authoritative transportation agency, and we have done desensitization due to confidentiality reasons.

**Figure 1:**
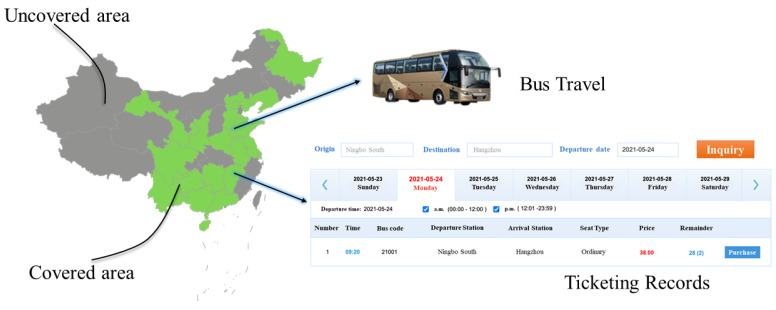
The bus ticketing data covered 19 provinces, including 2705 long-distance bus station’s travel records. The data range from April 2020 to August 2020. Each record describes the detailed information of a passenger’s trip, including start time, arrival time, name of the departure station, name of the terminal station, and the passenger’s gender and ID.

The bus ticketing data are gathered through multiple approaches from different bus systems. The collection is extremely cumbersome. Our data involved in 19 provinces across China, which is the largest range that can be reached so far. The data situation is shown in Figure 1. Green indicates the area covered, and gray indicates the uncovered area. Note that these 19 provinces refer to the province where the departure station is located.

Bus ticketing big data includes 2705 long-distance bus station’s travel records. The data range from April 2020 to August 2020. Each record describes the detailed information of a passenger’s trip, including start time, arrival time, name of the departure station, name of the terminal station, and the passenger’s gender, age, and ID.

It is worth noting that there are ticket records involving Taiwan and Hainan through the location matching in Section 2.2.2 and Section 2.2.3. This subdataset is ferry ticketing, but it accounts for less than 5% of the entire dataset, which has almost no impact on our research results and can be ignored.

Baidu API provide location information of provinces and cities, convenient for drawing trajectory map and station passenger flow bubble chart.

### 2.2. Data Preprocessing

Our overall computing framework is Spark. The bus ticketing data contains ten variables. Taking Shandong Province as an example, the original data are shown as Table 1.

The Table 2 showed the meanings of the ten variables:

SeatType is almost irrelevant to the problem we want to investigate, so we choose to delete this variable. Then we deleted the station ID and reserved the station names. Because our data comes from multiple bus systems, and the codes of different systems are different. The coding standards are not uniform. So we use the station name as the station’s identity.

#### 2.2.1. Missing Value Processing

**Figure 2:**
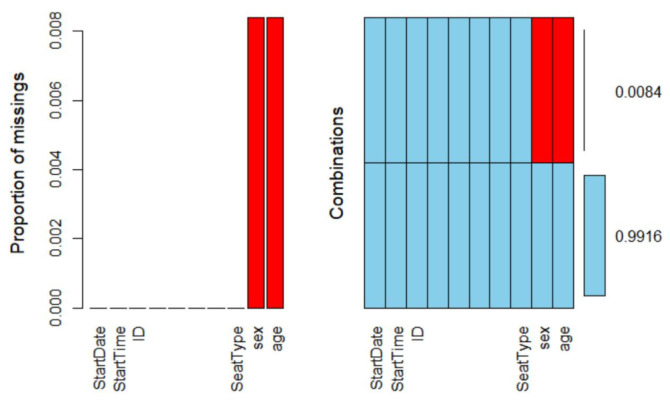
Variable outlier detection chart. Only the variables Sex and Age have missing values.

It can be seen from the Figure 2 that the missing values in the data are all on the two variables: age and sex.

The variables Sex and Age have missing values, and there is a synergy between them, they all have 837,877 missing items.

Records containing missing values account for less than 1% of the total. We found that the missing data only appeared in Sex and Age and appeared in pairs. We carefully investigated the reasons for this situation and traced back to before data desensitization. We found that the lack of Sex and Age of some passengers was caused by the purchase of tickets using passports and other identification certificates. We cannot accurately estimate the Sex and Age value of these passengers, so the interpolation method is not appropriate and may cause deviations in the results. Therefore, we finally choose to delete the incomplete records directly.

#### 2.2.2. Match Station Location Information

Use StartStationName and ReachStationName to match provinces and cities.

StartStationName corresponds to the outflow, and ReachStationName corresponds to the inflow. We find that the outflow and the inflow are relatively equal in the overall data set. So we chose to use StartStationName to match the location information, and the outflow was the main research problem.

We use string extraction to get the city from the station name.

Note this feature: most records contain specific cities and provinces. Only a few records do not contain clear location information (for example, “South Station”), and we deleted them directly.

#### 2.2.3. Exact Latitude and Longitude Matching

Then in order to obtain accurate location information, we use Baidu Map API to get the latitude and longitude of the station.

We use the R package “baidumap” to connect to the Baidu Map API and obtain the key through the Baidu Map open platform to extract the latitude and longitude of the region. Among them, data processing and using API to retrieve regional longitude and latitude operations are implemented through parallel computing, which explicitly involves the contents of packages such as “foreach”, “parallel”, and “future apply”.

**Figure 3:**
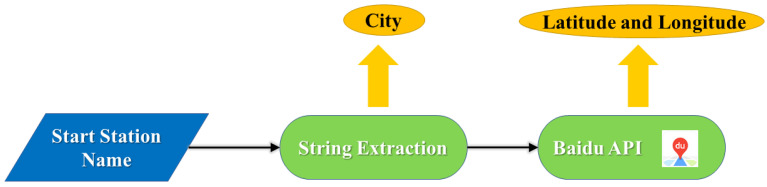
Location matching progress.The main steps are the extraction of cities and the acquisition of latitude and longitude.

Our location matching work can be clearly shown in Figure 3:

### 2.3. Technical Design Strategy: Region Division

**Figure 4:**
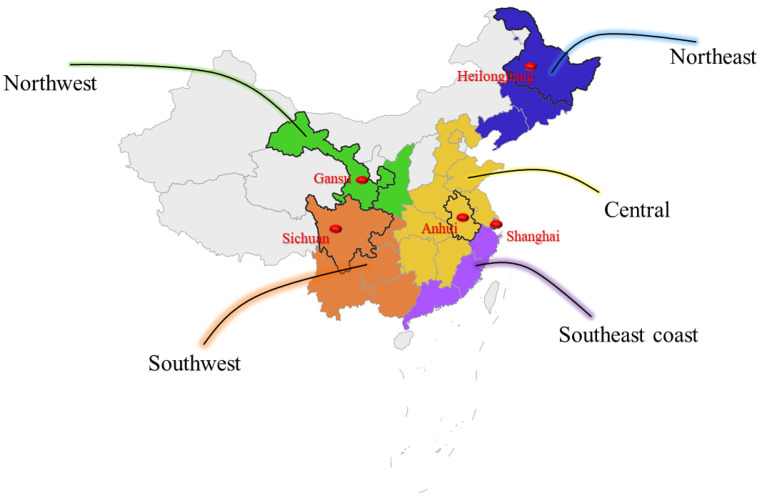
Classified provinces based on geographical status and economic conditions. Then select five typical provinces.

To make the chart not messy and obtain the effective results, we classified provinces based on geographical status and economic conditions. The geographical status is the main administrative division standard in China, hence we divided 19 provinces into five regions: the northeast, northwest, southwest, and southeast coastal regions. Shanghai, Guangdong, and other provinces are economic development regions and important labor input regions for the southeast coastal areas. The central regions represented by Henan, Jiangxi, Anhui, and other provinces, are Chinese labor export provinces. Northwest areas geographical location is relatively remote and the population flow is not as large as the provinces in the central and southern regions. Provinces divided in the same category have similarities in economic and living phenomena. We believe that it is practical to investigate the travel behaviors based on this division. The region division is shown in Figure 4.

The 19 provinces are divided into five regions:

(1) Northeast: Heilongjiang, Liaoning;
(2) Northwest: Gansu, Shanxi;
(3) Southwest: Guizhou, Sichuan, Yunnan, Guangxi;
(4) Southeastern coastal: Shanghai, Jiangsu, Guangdong, Hainan;
(5) Central: Hunan, Henan, Jiangxi, Anhui, Beijing, Shandong, Hebei.

We use Baidu Map API data to obtain latitude and longitude information. Considering the quota restrictions of Baidu Maps API, we have selected a province as the representative of the region and minimized the influence of other interference factors, making them more geographically meaningful, based on the above division of regions.

The southeast coastal area is represented by Shanghai because Shanghai is located at the estuary of the Yangtze River and is a first-tier city in China, which can well represent the economically developed coastal areas. Anhui represents the central region. Anhui Province does not have too extreme indicators. It is not a province with tourism as its primary pillar industry, nor does it along the coast. Heilongjiang represents the northeast region, the geographical location is representative, and there are no other interference features such as coastal areas, tourism, or colossal population size. The northwest region is represented by Gansu, which has more prominent geographical features than Shaanxi Province. The southwest region is represented by Sichuan Province, which minimizes interference factors such as the nature of tourism and the nature of ethnic minority settlements.

**Figure 5:**
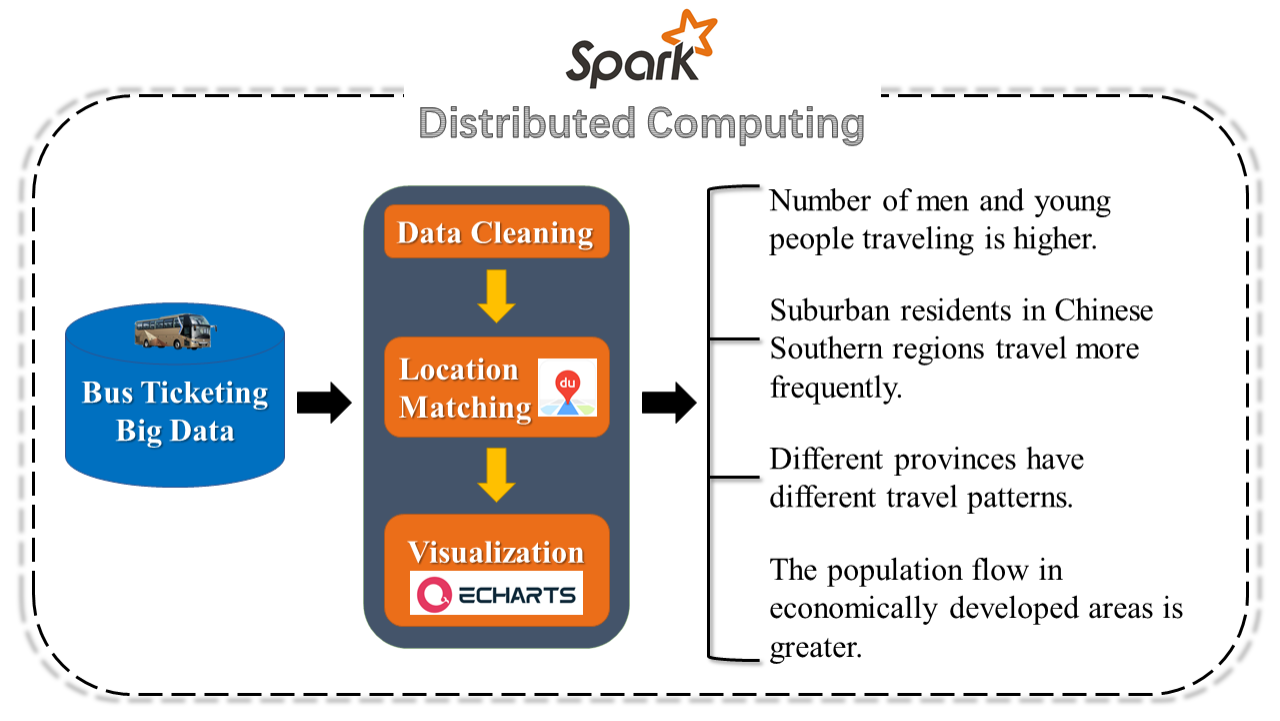
The Research Framework. The calculation framework, processing flow and main results are shown.

In summary, the conceptual framework of our research can be concisely expressed with a flow chart (Figure 5).

## 3. Results

### 3.1. The Impact of Regional Differences on Travel Behavior

We conducted frequency statistics on travelers in 19 provinces from April 2020 to August 2020 and regarded them as high-frequency, medium-frequency, and low-frequency groups. People who have traveled no more than 10 times are low frequency, 10–45 times are medium frequency, and those who have traveled more than 46 times are high frequency. We finally got the following results (Table 3).

Compared with the northern regions, the proportion of low-frequency populations in the southern and central regions has experienced a cliff-like decline.

The northeast and northwest regions include: Heilongjiang, Liaoning, Gansu, and Shaanxi; the southwest, southeast, and central regions include: Guizhou, Sichuan, Yunnan, Guangxi, Shanghai, Jiangsu, Guangdong, Hainan, Hunan, Henan, Jiangxi, Anhui, Beijing, Shandong, Hebei. We found that people in areas with frequent economic activity and high population density are more likely to travel. This is different from the travel pattern in the early stages of the COVID-19 epidemic. In the early stages of the COVID-19 epidemic, people’s travel frequency was relatively low due to various lockdown policies and travel restrictions. Even in some countries and regions, population mobility tends to almost zero [31,32]. We speculated that in the post-COVID-19 phase, people have begun to make necessary travels due to various reasons such as life requirements with the lifting of travel restrictions. However, we have not collected enough evidence to verify this speculation. Perhaps this requires further research through richer data and information.

### 3.2. The Impact of the COVID-19 on Travel Behaviors of Different Genders and Ages

**Figure 6:**
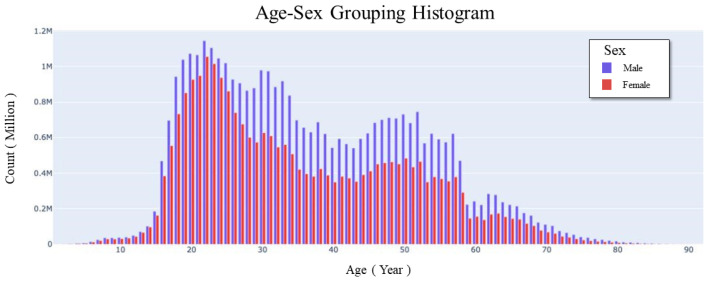
Gender and age statistics of travel population. The abscissa is age, and the ordinate is the number of travellers. The blue column represents males and the red column represents females.Regardless of male or female, there is a small peak in the graph when the age is about 20. The age range of 18 to 26 has a large number of passengers. The men are most distributed between the ages of 20 to 58 years old. Compared with men, females’ age distribution is more concentrated, the number of age of 20 is the largest.

The analysis of characteristics of passenger is based on data from April 2020 to August 2020. The results are shown in Figure 6.

The number of male passengers is higher than that of female passengers, and there are more young and middle-aged people. There are two peaks at the age of 20 and the age of 50. According to the Sixth Census, the migrants with the highest proportion is people aged 20–24, the second highest is who aged 25–29, and the third is 15–19 and 35–39. Worth noting that the results we got are very similar to the statistical report [28] made by China’s authoritative transportation agency in 2013.

The report [28] clarified that commuting is the most important part of bus travel. Then we associated that the commuters are mainly young and middle-aged passengers and companies have resumed work. The government also lifted travel restrictions. So we reasonably speculate that the current travel pattern has almost returned to normal due to the current travel pattern is very similar to the travel pattern before the COVID-19 epidemic.

Furthermore, with the increase of age, a new travel mode gradually appears. It is shopping-tourism immigration. Numerous works of literature [33,34] have shown that people’s travel enthusiasm increases linearly with age. China lifted travel restrictions in July 2020, resulting in a significant increase in the number of shopping-tourism trips for the elderly. It also explains why there will be a new peak of travel at 50 years old.

### 3.3. Typical Region Travel Trend Analysis

**Figure 7:**
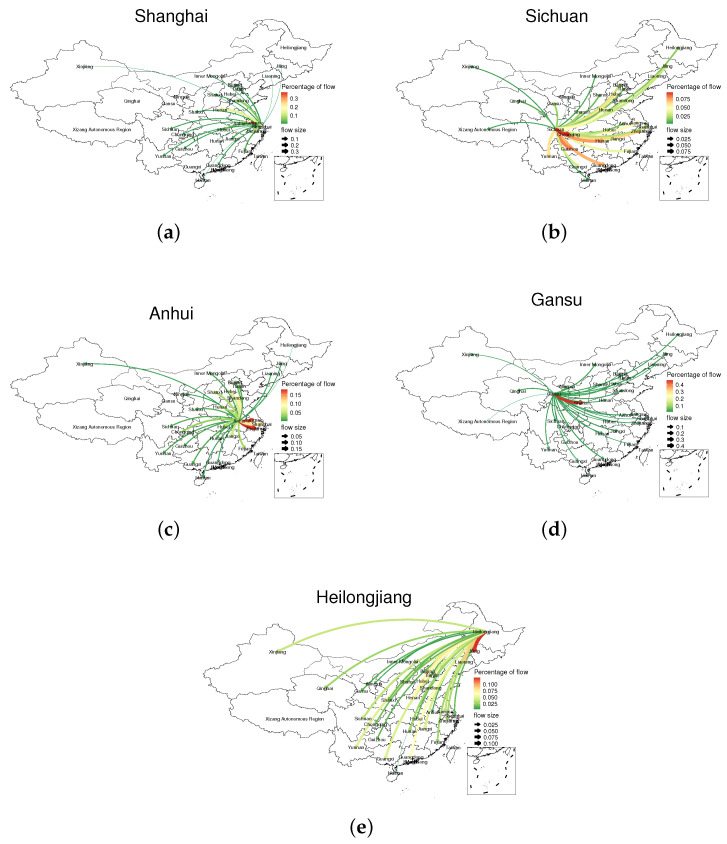
Travel flow map. The color of the arrow indicates the amount of passenger flow. Red indicates high passenger flow, and green indicates low passenger flow. The arrow also indicates the direction of passenger flow and the thickness of it corresponds to the amount of the flow. Note: all passenger flows are converted into percentage form, and the value range is from 0 to 1. (**a**) Passenger flow from Shanghai to other provinces. (**b**) Passenger flow from Sichuan to other provinces. (**c**) Passenger flow from Anhui to other provinces. (**d**) Passenger flow from Gansu to other provinces. (**e**) Passenger flow from Heilongjiang to other provinces.

We focused on the bus passenger flow in the five typical regions. The proportion of the internal circulation flow of each province is shown in the Table 4, and the flow of the migrant population flow is shown by the flow map (Figure 7).

This part of the research is based on data from April 2020 to August 2020.

We found that Gansu Province has the highest proportion of trips within the province, as high as 30%. However, Shanghai’s internal passenger flow only accounts for 4.55%. This is due to China’s economic structure and the nature of the city. Shanghai is a metropolis with frequent exchanges of people and a well-developed economy. Many workers choose to work in Shanghai, but live in the surrounding areas to reduce the cost of living. Bus commuting is their main method [28]. The remaining provinces also have 10% to 20% internal mobility, but we pay more attention to population migration outside the province, which has a greater impact on the spread of the epidemic and the stability of the country.

We also calculated the number of non-duplicated routes in the five regions to reflect the diversity of bus travel in different provinces (Table 5).

Combining tables and figures, we obtained some valuable information and inferences.

Shanghai residents have the least number of travel routes, and their destinations are also relatively close overall. Most of them move to the periphery of Shanghai and the central and eastern regions. We found that Shanghai’s travel structure is closely related to Shanghai’s social life. As an international metropolis, Shanghai is a vital labor import province, so there is less large-scale labor export. The city population is more inclined to stay in the city. Even if it needs to move, taking into account living and cultural habits and other reasons, most of the land is in other eastern cities, especially other first-tier cities. They are more inclined to go to surrounding provinces and have simple travel routes.

The travel range about Sichuan Province is wide. The main destinations are the Sichuan-Chongqing region, Yunnan, Guizhou, Hunan, Guangdong, and the Pearl River Delta region. Most of them are in the southwest and southeast coastal areas. Sichuan Province is a populous province. In 2018, the total population of Sichuan Province reached 83.41 million. Sichuan Province is located in the inland area of southwestern China. It can be seen from the figure that its population travel destinations are concentrated in the Sichuan-Chongqing region and the southeast coastal area. The groups of receiving the floating population in Sichuan Province are Shanghai city, Shenzhen city, and Chongqing. Sichuan Province is a vital labor export province and a talent supply base in China. Therefore, most of the migrant population in Sichuan Province flows to areas with high demand for talents and sufficient labor positions. At the same time, the number of students in Sichuan Province is vast, and people tend to move to areas with more developed economies in order to pursue high-quality educational resources, which explains why a lot of people move to Shanghai, Guangzhou and other southeast coastal areas.

Except for itself, the passenger flow gathering areas from Anhui Province are mainly in Jiangsu, Zhejiang, and Shandong province. Anhui Province is also a relatively populous province in China, and its total population reached 63.240 million in 2018. Although the latitudes of Anhui Province and Sichuan Province are almost the same, their travel trajectories are quite different. First, Anhui Province residents are more inclined to move to Jiangsu and Henan provinces adjacent to Anhui and have a lower preference for other coastal provinces in the east. The fact is that Anhui, Jiangsu, and Zhejiang have formed a stable economic ecosystem [35]. They have similar economic behaviors and frequent economic activities, and their close trade exchanges have made their population convection the most frequent. Secondly, although the number of travel trajectories in Anhui Province is significantly more than that of Sichuan, the richness and coverage of travel trajectories in Anhui Province are not as good as those in Sichuan. It can be seen that the travel choices of residents in Anhui Province are homogeneous, and the activity is negligible. They lack the motivation to fight outside like Sichuan.

The passenger flow of Gansu Province spreads to all parts of the country, but it is mainly concentrated in Gansu and Shaanxi provinces. Gansu Province has a very wide range of travel, due to its geographical environment and historical legacy. Gansu is a relatively poor province in China, which prompted residents to migrate to other provinces for pursuing a better life.

We found that the Heilongjiang population has no apparent preference for travel. From north to south, most of China is involved, but its small gathering areas are mainly distributed near first-tier cities such as Beijing, Shanghai, and Guangzhou. It is consistent with their pursuit of better economic conditions and living environment.

In summary, in the post-epidemic era, people’s travel patterns are mainly influenced by various regions’ economic behaviors and cultural habits. Under the conditions of full implementation of resumption of work and production and liberalization of travel restrictions, people’s travel patterns are on the right track. The interference of the epidemic has been greatly reduced.

### 3.4. Passenger Flow Main Flocking Areas in the Post-COVID-19 Phase

**Figure 8:**
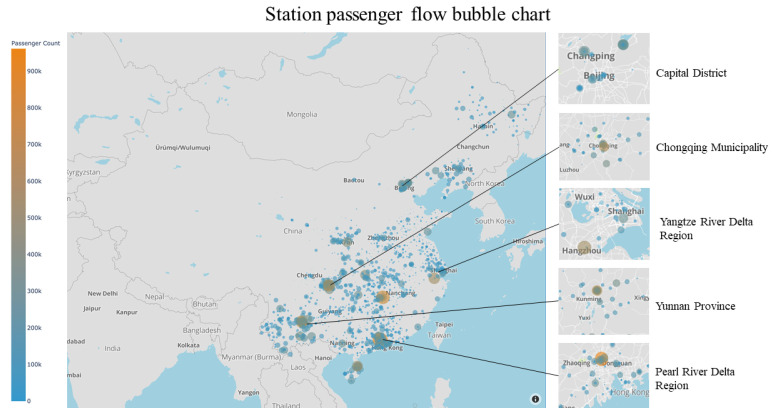
Station distribution map. The size and color of the bubble indicate the amount of the passenger flow. The orange bubble indicates that the passenger flow of the station is large, and the blue is low. Most of the bubbles are concentrated in Yangtze River Delta, the Pearl River Delta, Beijing, Chongqing, and Yunnan Province. There are several prominent big and orange bubbles in Guangzhou, Changsha, Kunming and Hangzhou, indicating that the passenger flow of these stations is much higher than that of other stations.

We drew a bubble map of the passenger flow of all bus stations in the dataset (Figure 8).

The chart is based on data from April 2020 to August 2020.

Several stations with significant passenger flow are the leaders of China’s top ten passenger bus stations. The main passenger flow areas are in the Yangtze River Delta, the Pearl River Delta, Beijing, and Yunnan Province. The inflection point of the epidemic in China appeared in March, and in April, May, and June, it achieved full resumption of work and production. In July, the Ministry of Culture and Tourism issued a notice to loosen travel restrictions, which made the move across regions even more accessible, especially in labor-intensive areas such as the Yangtze River Delta and the Pearl River Delta and significant tourist provinces led by Yunnan Province.

## 4. Discussion

This research uses approximately 26,000,000 bus ticketing records across 19 provinces of China from April 2020–August 2020 for 2705 stations to investigate the population flow of Chinese suburban residents in the post-COVID-19 phase. Suburban residents are mostly middle and low income groups. The statistical report of the authoritative transportation agency in China shows that bus passengers indeed have the characteristics of suburban residents. This is due to China’s residential structure and bus route arrangement principles.

The methodology used in this paper is mainly descriptive statistics. The 19 provinces involved in the dataset are divided into 5 regions depending on the geographical status and economic level. We first analyzed the travel frequency in different regions and found that people still travel mainly in low and medium frequencies, but the travel frequency is extremely different in the southern and northern regions. The travel frequency is generally higher in the southern region, and the number of middle and high frequency travellers is about 15% higher than that in the northern region. Secondly, we studied the gender and age characteristics of the travel population, and found that men and young people are more likely to travel, with more travellers and higher frequency. Subsequently, to explore the differences in travel behaviors and patterns brought about by regional differences, we selected five typical regions to draw travel flow maps to observe their destination preference and travel trends. We found that labor exporting provinces are more inclined to go to economically developed areas, such as Beijing, Shanghai and other first-level cities; while the inclined areas of economic developed area like Shanghai is relatively small and mainly concentrated in surrounding areas. It may be due to the fact that the economic developed areas have formed a stable economy ecosystem, whose population mainly comes from the surrounding areas, such as living in the surrounding areas and working in the central city.

Associate with previous literature, we find that the higher the economic level, the more active the social activities are, the easier it is for residents to travel. Men and young people are more likely to travel and in high-frequency as the main labor force. Using five typical regions as verification, we found that it is indeed the case. We also found that Anhui, Henan, and Jiangsu have formed a relatively stable economic ecosystem, and their population flows almost circulate internally. The travel trajectories in Shanghai and other economically developed areas are simple, and travel destinations are concentrated in partial, mainly in the surrounding areas. Major labor supply provinces such as Heilongjiang and Sichuan have diverse and complex travel trajectories throughout China, but tend to flow to economically developed regions.

Our results indicate that suburban residents travel behaviors are affected profoundly by economy and consistent with the inherent behavior patterns before the COVID-19 outbreak. The current travel pattern is very similar to the travel pattern before the COVID-19 epidemic [28]. Maybe we could reasonably speculate that the current travel pattern has almost returned to normal. Suburban residents travel behaviors has been seldom affected by pressure from medical resources [36], government policies and travel panics. It’s not as conservative as we thought, the travel behaviors of suburban residents in the post-COVID-19 has become casual, which proves that people consider that the surrounding environment is relatively safe, and the probability of spreading the epidemic has dropped to a very low level.

Our research makes up for the lack of apposite data among suburban residents, and helps people understand the travel behaviors and patterns of suburban residents more scientifically and reasonably in the post-COVID-19 phase. The research is significant to protect the achievement of fighting the COVID-19 epidemic and maintain the national stability.

Our research lacks data on the situation before the COVID-19 pandemic. It is an objective limitation because this collection and statistics work only started in the late stages of the epidemic. The nationwide aggregation work has not been done before. So we can only discuss and analyze our results by referring to previous research conclusions and using their conclusions as a hypothesis. Therefore, we can only characterize how people are traveling in the post-COVID-19-phase and get some reasonable speculations. If possible, we hope to get more data and richer information to conduct deeper research. It is the direction we need to work hard on.

It is also worth to mention that our research only investigated bus travel, which is a part of the public transportation system. Some suburban residents may also travel by plane, train, or car, and therefore our research results can only explain the characteristics of most suburban residents. Moreover, no data were available about the supply regarding bus line services, which is a physical limitation because there is no nationwide networked bus system providing supply information for all bus lines. Under such circumstances we can only use as extensive as possible data to cover as many as possible areas and routes, and we also lack specific data on some regional restriction policies to do more advanced research. These defects may require more data and information to improve.

## Figures and Tables

**Table 1:** Taking Shandong as an example to display the dataset. We only intercepted the first 10 lines.

Start Date	Start Time	ID	Start Station ID	Start Station Name	Reach Station ID	Reach Station Name	Seat Type	Sex	Age
20200804	63000	58091896	370502001	Dongying Station	782	Qingdao North	1	0	40
20200804	63000	12353227	370502001	Dongying Station	782	Qingdao North	1	0	13
20200804	130000	95292886	370502001	Dongying Station	401	Huangdao	1	0	26
20200804	130000	99522183	370502001	Dongying Station	401	Huangdao	1	0	51
20200807	130000	30619858	370502001	Dongying Station	401	Huangdao	1	1	23
20200805	81000	23133430	370502001	Dongying Station	1012	Hezeguolu	1	0	22
20200803	135000	6097925	370502001	Dongying Station	492	Jiaozhou	1	0	22
20200804	73000	74505130	370502001	Dongying Station	438	WeiFang	1	1	22
20200803	133000	45878620	370502001	Dongying Station	879	Tianjin	1	0	30
20200803	133000	26409453	370502001	Dongying Station	879	Tianjian	1	1	29

**Table 2:** Variable description. The dataset include 10 variables.

Variable Name	Meaning
StartDate	Departure date
StartTime	Departure time
ID	ID of passenger
StartStationID	ID of start station
StartStationName	Name of start station
ReachStationID	ID of reach station
ReachStationName	Name of reach station
SeatType	The type or the level of the seat
Sex	The gender of the passenger; 0-female; 1-male
Age	The age of the passengger

**Table 3:** Trip frequency statistics. Northwest China and Northeast China are relatively similar, with the low-frequency population reaching 90%, medium-frequency population being 5% or less, and high-frequency population being less than 1%; Southwest, Southeastern coastal areas, and central regions showed similar results, with low-frequency populations at about 76%, medium frequency populations at about 20%, and high-frequency populations at about 3%.

Rigion	Low Frequency	Medium Frequency	High Frequency
Northeast	93.98%	5.11%	0.91%
Northwest	95.69%	3.71%	0.60%
Southwest	76.06%	20.73%	3.21%
Southeast Coast	76.04%	21.03%	2.93%
Central	76.06%	20.73%	3.21%

**Table 4:** The proportion of the passenger flow in internal circulation in five provinces.

Province Name	Shanghai	Heilongjiang	Gansu	Sichuan	Anhui
Flow in internal province	4.55%	14.73%	30.63%	10.41%	22.85%

**Table 5:** Non-duplicate routes count. Statistical results of non-duplicated paths in different regions. Heilongjiang and Anhui Province have the largest number of routes, and Shanghai has the least number of routes.

Province Name	Shanghai	Heilongjiang	Gansu	Sichuan	Anhui
Different Routes Count	1257	6437	2348	4742	6472

## Data Availability

The data are not publicly available due to the data relates to the record of a large number of people traveling by bus in China during the post-COVID-19 phase. These records are officially undisclosed.

## Abbreviations

The following abbreviations are used in this manuscript:	
COVID-19	Coronavirus Disease 2019
API	Application Programming Interface
ID	Identification
